# Supplementary figures and images for: Reward-driven changes in striatal pathway competition shape evidence evaluation in decision-making
Source: PLoS Comput Biol. 2019 May 6;15(5):e1006998. doi: 10.1371/journal.pcbi.1006998 (PMC6534331; doi:10.1371/journal.pcbi.1006998)

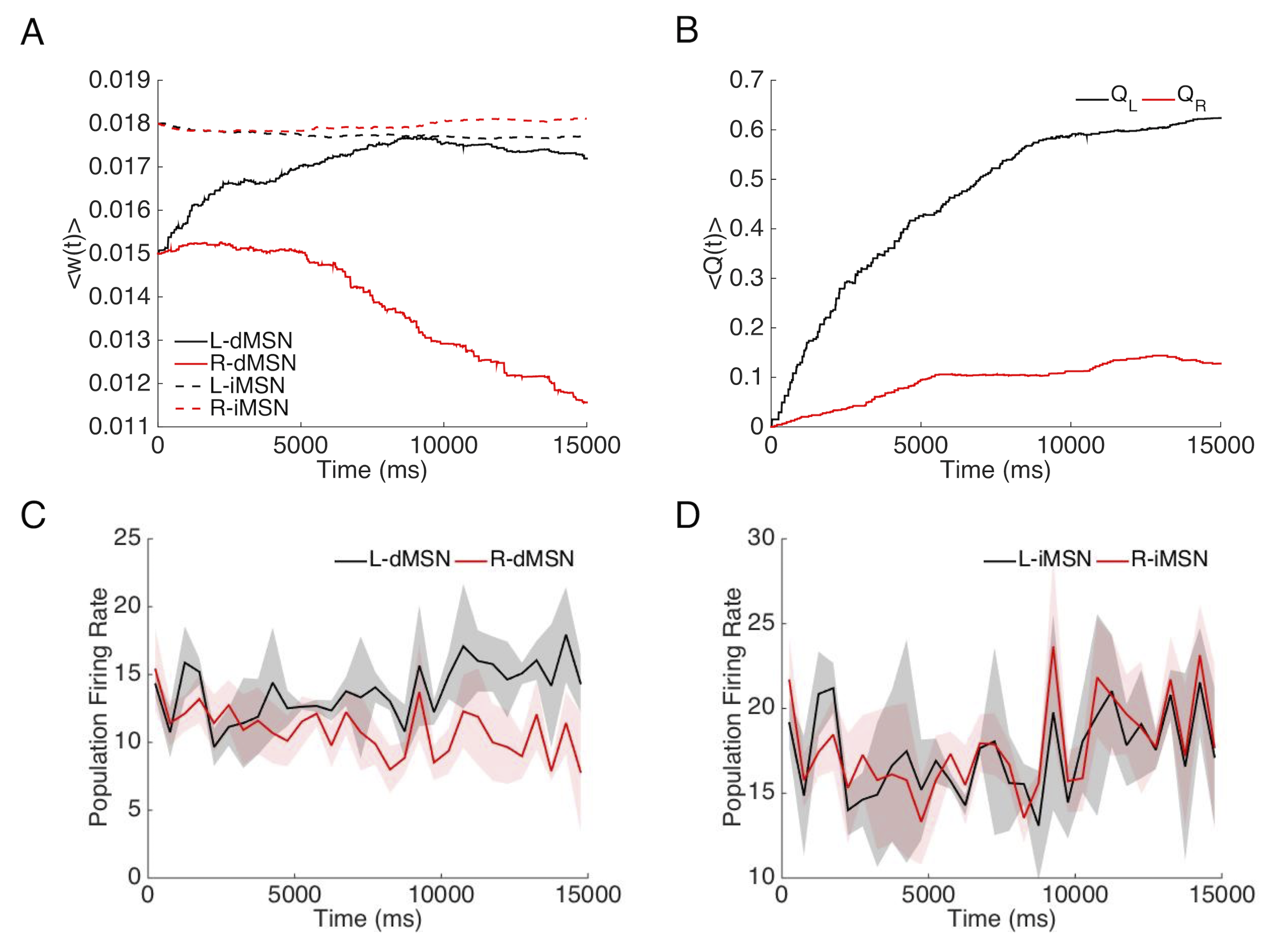

Supplement: S1 Fig — A: Averaged weights over 7 different realizations and over each of the four specific populations of neurons, which are dMSN selecting action L (solid black); dMSN selecting action R (solid red); iMSN countering action L (dashed black); iMSN countering action R (dashed red). B: Averaged evolution of the action values QL (black trace) and QR (red trace) over 7 different realizations. C: Average neuronal firing rate (spike count within a time bin divided by bin duration) across neurons in the dMSN population selecting action L (black) and R (red), respectively, over time. D: Firing rates of the iMSN populations countering actions L (black) and R (red) over time. Data in C,D was discretized into 50 ms bins. The transparent regions depict standard deviations. (TIF) [file pcbi.1006998.s001.tif]

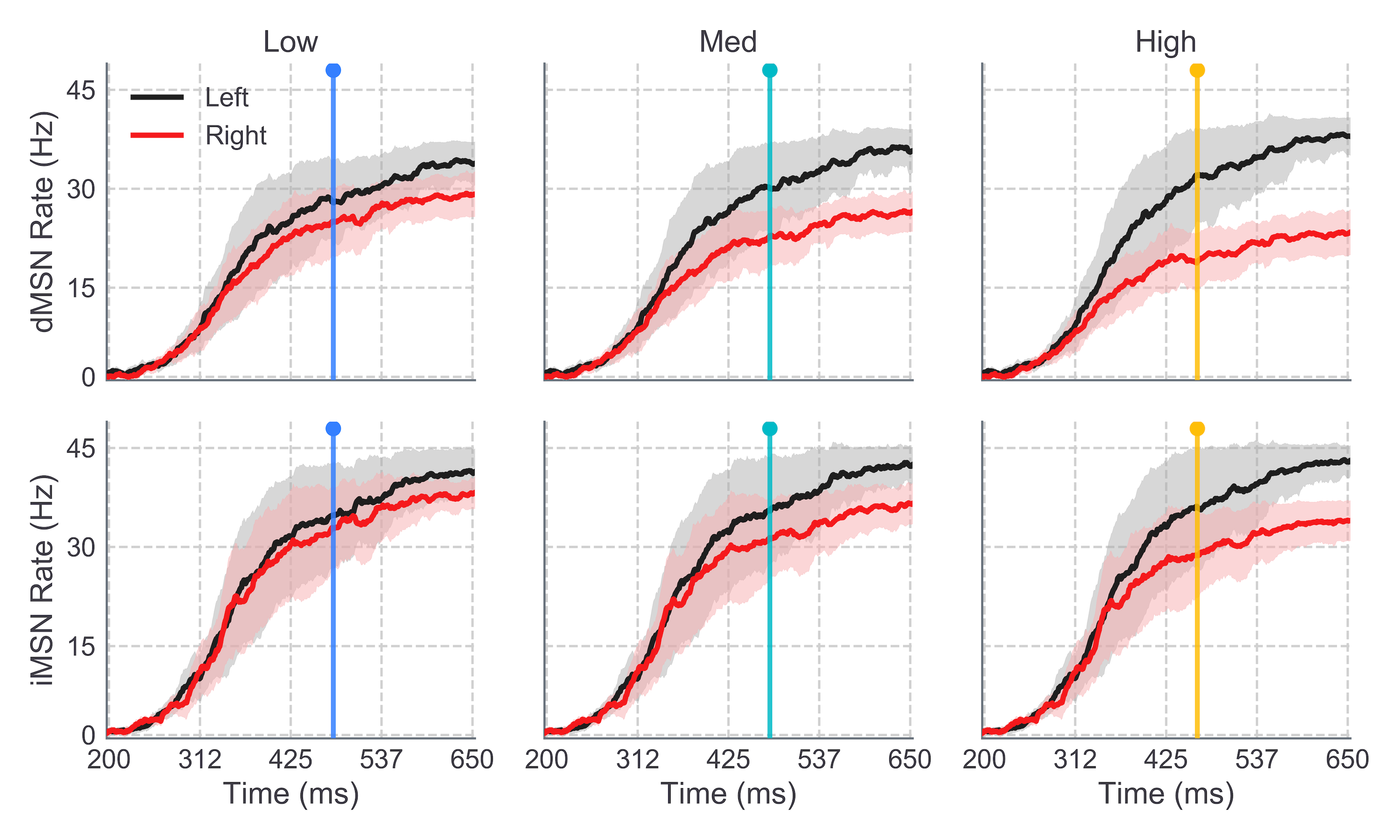

Supplement: S2 Fig — Timecourse of average firing rates of dMSN (upper) and iMSN (lower) populations in left (black) and right (red) action channels are shown for low (left), medium (middle), and high (right) reward conditions. (TIF) [file pcbi.1006998.s002.tif]

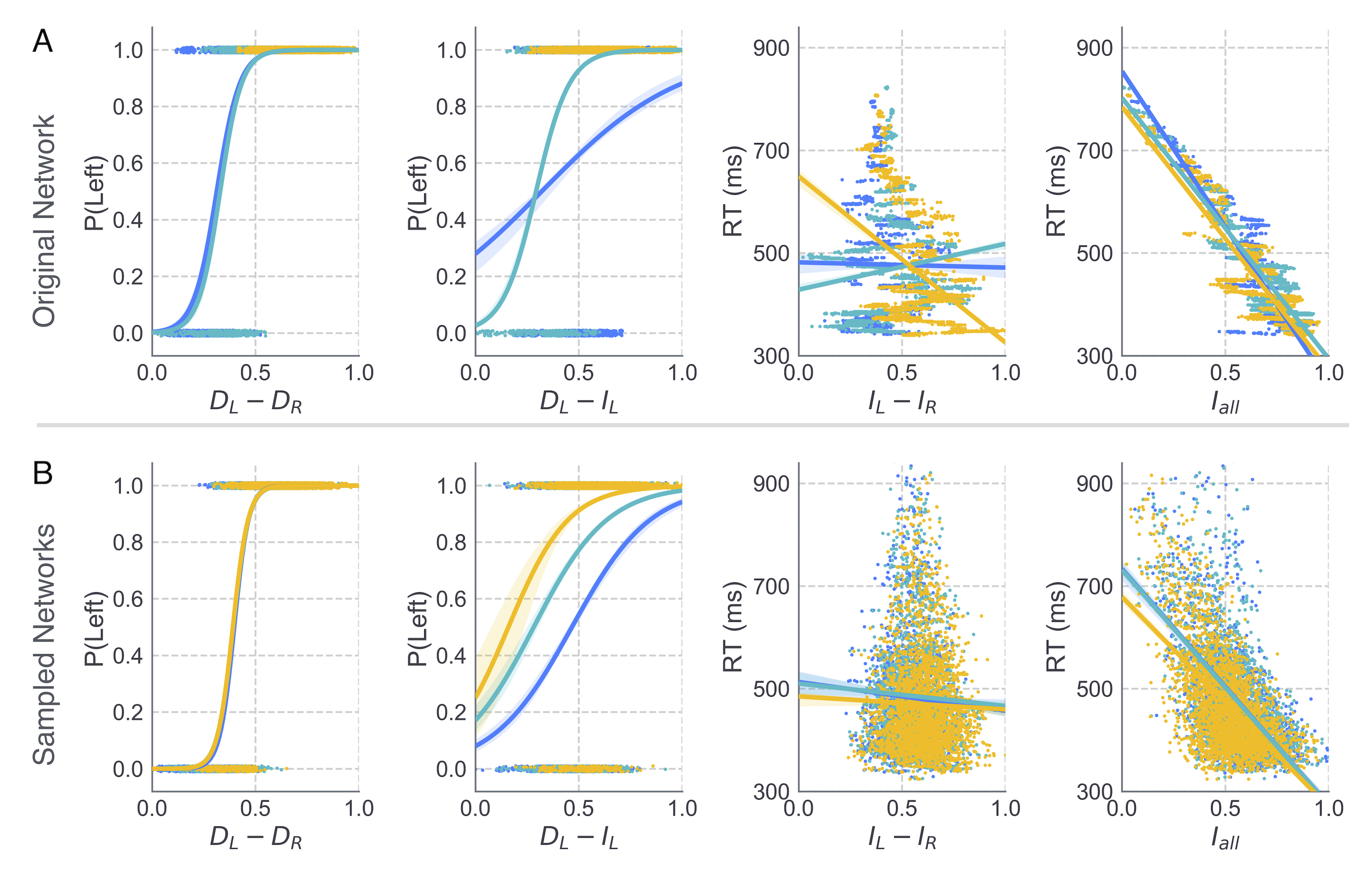

Supplement: S3 Fig — A. Simulated decision outcomes (left, center-left) and RTs (right, center-right) from the original CBGT network are plotted as a function of the four striatal summary statistics used as predictors of trialwise changes in DDM parameters. Choice outcome is plotted as a function of direct pathway measures, DL − DR (left) and DL − IL (center-left); RT as a function of IL − IR (center-right) and Iall (right). Each dot represents data from an individual trial in the low (blue), medium (cyan), and high (yellow) reward conditions. Logistic (left, center-left) and linear (right, center-right) regression predictions for each condition are shown as lines. B. The same data as shown in panel A from multiple (N = 15) randomly sampled CBGT networks. (TIF) [file pcbi.1006998.s003.tif]
